# Snake richness in urban forest fragments from Niterói and surroundings, state of Rio de Janeiro, southeastern Brazil

**DOI:** 10.3897/BDJ.4.e7145

**Published:** 2016-02-04

**Authors:** Nathalie Citeli, Breno Hamdan, Thais Guedes

**Affiliations:** ‡ Laboratório de Coleções Biológicas e Biodiversidade, Instituto Vital Brazil, Rua Maestro José Botelho, 64, 24230-410, Niterói, Brazil; § Laboratório de Biologia Evolutiva Teórica e Aplicada, Departamento de Genética, Instituto de Biologia, UFRJ, Ilha do Fundão, Rio de Janeiro, Brazil; | University of Gothenburg. Department of Biological and Environmental Sciences. Carl Skottsberg gata 22B, Se 405 30, Göteborg, Sweden

**Keywords:** Urban Inventory, Serpentes, Species Richness, Forested Areas, Endemism, Conservation

## Abstract

**Background:**

The Atlantic Forest is a hotspot for biodiversity, an area which houses high species richness and endemism, but with high level of threat. With reference to the herpetofauna, until recently there has been no detailed information regarding diversity of snakes recorded in the Atlantic Forest, the number of endemic species and their distribution ranges. While these basic data were missing, large areas of original forest have continued to be lost to increased urbanization and human population, representing a severe threat to the biodiversity.

**New information:**

We recorded 28 snake species in our study area. Dipsadidae was the richest family with 14 species, followed by Colubridae (eight species), Boidae (two species), Viperidae (two species), and Anomalepididae, Elapidae and Typhlopidae (one species each). Most species were forest inhabitants (61%), of which 13 are endemic to the Atlantic Forest. There were no clearly defined species clusters regarding species composition. None of the species recorded in Niterói are listed as threatened in the Brazilian Redlist. However, most of them are strongly associated with forested areas and, perhaps, are not adapted to live in small fragments. Thus, more initiatives should be implemented to evaluate the true conservation status of these species in order to better protect them.

## Introduction

The Brazilian Atlantic Forest is one of the largest Neotropical rainforests originally covering around 150 million ha (Tabarelli et al. 2005, Ribeiro et al. 2009). This forest has one of the highest species richness and endemism levels on Earth (Myers et al. 2000). However, it is highly threatened (Ribeiro et al. 2009) which, along with its high endemism, makes it a biodiversity hotspot for conservation (Myers et al. 2000). Recent data suggested that the Atlantic Forest has already lost more than 84 to 89% of its area, while the remaining 100,000 km^2^ comprises only small isolated fragments (Tabarelli et al. 2005, Ribeiro et al. 2009). These fragments may still house many unknown species, with several new snake species having being described recently (Lewinsohn and Prado 2005, Passos et al. 2010, Pires et al. 2014).

The future of the biodiversity in the Atlantic forest is under threat, since deforestation and habitat fragmentation impose barriers for individual migration, leading to the decrease and isolation of population and extinction in the long term (Tabarelli et al. 2005, Becher et al. 2007). The increase in urbanization and a growing human population, represent a cosiderable pressure to this area's biodiversity (Nations 2008). Niterói is a city on the coast of Rio de Janeiro, Brazil. About 23% of the state of Rio de Janeiro is covered by Atlantic Forest (SOS Mata Atlantica and INPE 2014). Recent studies have surveyed the herpetofauna of particular areas of the state and provided lists of snake species (e.g. Rocha et al. 2000, Rocha et al. 2004, Izecksohn and Carvalho-e-Silva 2001, Rocha and Van Sluys 2006, Almeida-Gomes et al. 2008, Almeida-Gomes et al. 2010, Pontes et al. 2009, Pontes and Rocha 2008, Vrcibradic et al. 2011, Martins et al. 2012), but urban areas remain poorly studied (Rocha et al. 2004). Certainly, no previous snake survey has been conducted in the municipality of Niterói.

Here we provide the first account of snake species richness in the urban area of Niterói. We compare our results with other surveys carried out in nearby areas in Rio de Janeiro and the Atlantic Forest. This study provides basic knowledge about diversity, geographic distribution, endemism, and ecology of species that could help in future conservation planning (Whittaker et al. 2005).

## Materials and methods

### Study area

**Figure 1.**
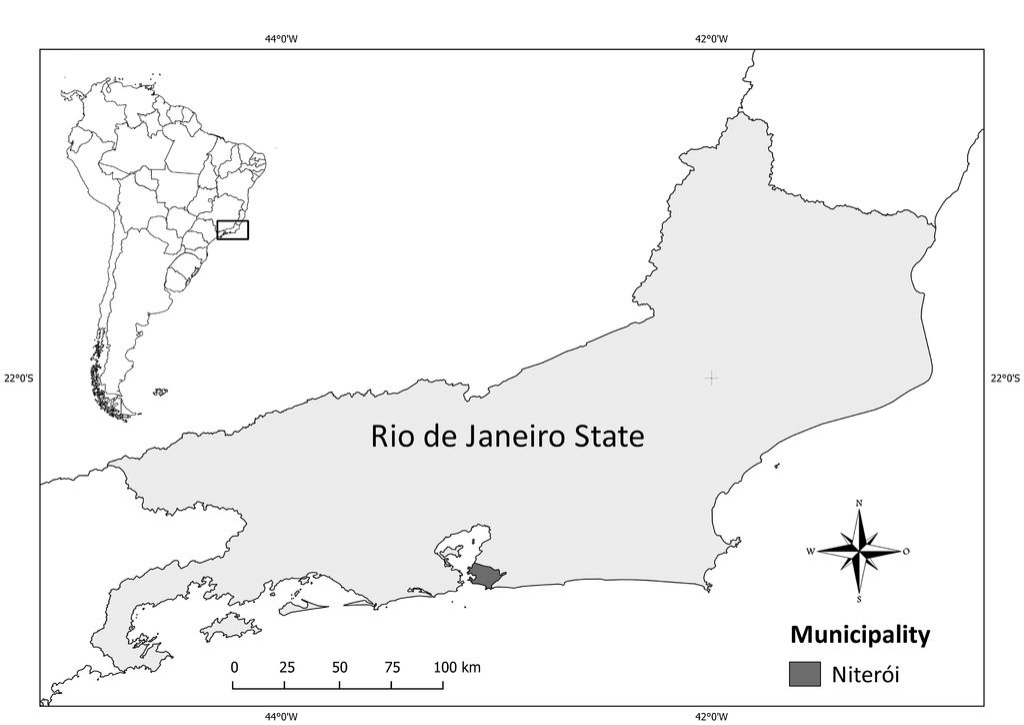
Map showing the localization of the municipality of Niterói in the Atlantic Forest Domain and in the state of Rio de Janeiro (featured area).

The city of Niterói (43°06'13"W, 22°53'00"S; Fig. 1) is currently the fifth most populous urban area of the state of Rio de Janeiro, housing about 500,000 inhabitants in an area of 133,000 km^2 ^(IBGE 2014). The city is located in the Atlantic Forest Domain, with altitude varying from sea level up to 412 m in the Elefante Mountain Range (CEPERJ 2013, Pontes 1987). About 99% of the city's original area was originally covered by the Atlantic Rain Forest, but vegetation cover today is reduced to about 23% (Ab'Saber 1977, Rizzini 1979, IBGE 1992, SOS Mata Atlantica and INPE 2014).

The climate in Niterói is of the Köppen’s type Aw Tropical, with high temperatures, rainy summers, and dry winters (Köppen 1936). Relative humidity is 79.1%, mean annual maximum and minimum temperatures are 27.3° C and 21° C, respectively, with extremes recorded of 10.1° C and 42° C (INMET 1992). The mean annual rainfall is 98 mm (Sentelhas et al. 2003) and the main sources of fresh water are the Macacu and Guapiaçu rivers (Águas 2010).

### Data collection

Species identification was based on the specimens examined by direct analysis of voucher specimens (Suppl. material 1) collected between 1956 and 2014 and housed in the following scientific collections: Museu Nacional da Universidade Federal do Rio de Janeiro (MNRJ), Coleção Científica de Serpentes do Instituto Vital Brazil (IVB), Coleção de Répteis da Universidade Federal do Rio de Janeiro (ZUFRJ), and Museu de Zoologia da Universidade de Campinas (ZUEC-REP). Museum acronyms are taken from Sabaj Pérez 2014, except IVB.

Specimens were examined by the authors and identified based on current and reliable taxonomic literature (e.g., Peters and Orejas-Miranda 1970, Peters 1960, Dixon and Kofron 1984, Dixon 1989, Dixon and Markezich 1992, Dixon et al. 1993, Franco and Ferreira 2002, Campbell and Lamar 2004, Hedges et al. 2014). The systematic arrangement followed Zaher et al. 2009 and nomenclature follows Costa and Bérnils 2014. We also provide additional information about habitat use and diet for each species based on data from the literature (Almeida-Gomes et al. 2008, Argôlo 2004, Gaiarsa et al. 2013, Hartmann et al. 2009, Marques et al. 2004, Vrcibradic et al. 2011, Pontes and Rocha 2008).

### Analysis

We performed a dissimilarity analysis with Jaccard’s coefficient and centroid clustering method of the species presence/absence matrix to compare our species list with others from the Atlantic Forest of the state of Rio de Janeiro. The analysis was implemented in the package *vegan* (Oksanen et al. 2015) of the R software (R Core Team 2014). The raw data used to perform the similarity analysis is provided in Suppl. material 2.

## Data resources

We recorded 28 snake species from 18 genera and seven families (see Checklist, Suppl. material 1). The most speciose family was Dipsadidae (14 species), followed by Colubridae (eight species), Boidae (two species), Viperidae (two species), Anomalepididae and Typhlopidae (one species each). The most common species found in the collections were *Bothrops jararacussu* (103 specimens), followed by *Bothrops jararaca* and *Micrurus corallinus* (78 and 32 specimens, respectively). The least common were *Chironius exoletus*, *Erythrolamprus aesculapii*, *Leptophis ahaetulla*, and *Liotyphlops wilderi* (one specimen each). We also established a first record from Niterói for *Clelia plumbea*, though it also seems to be very rare.

**Figure 2.**
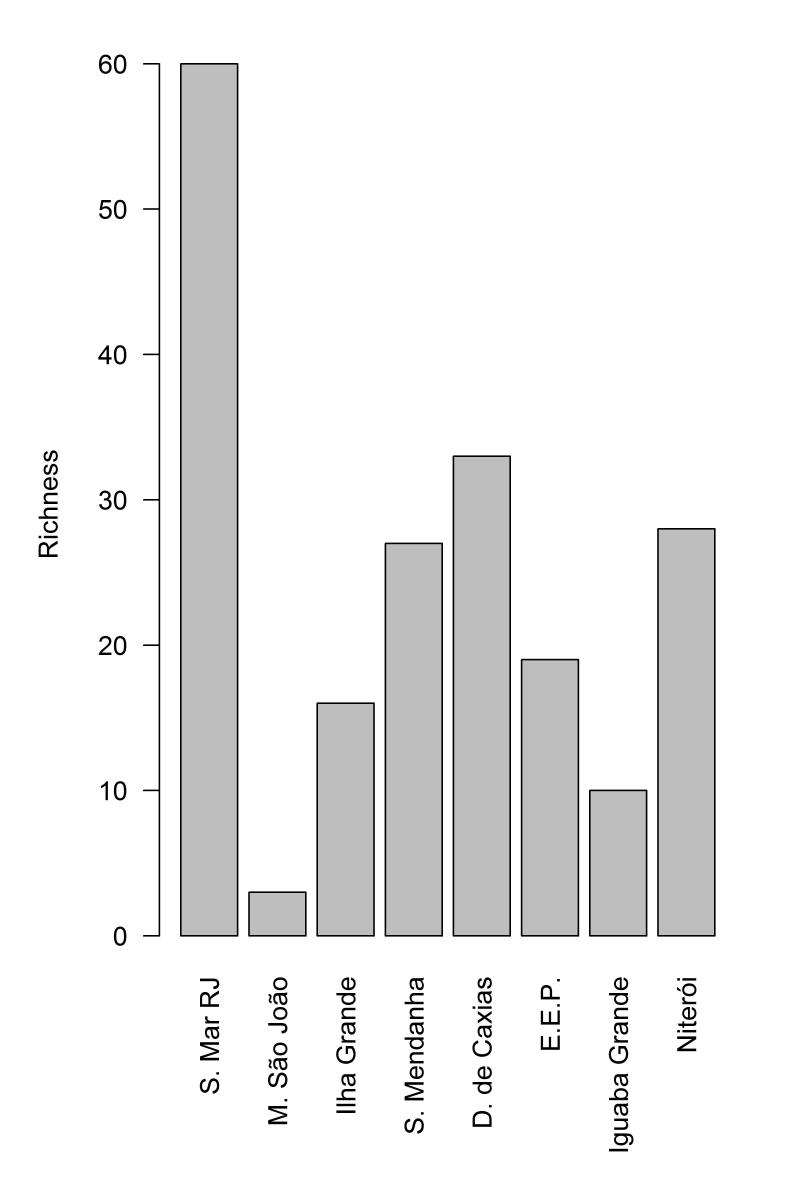
Richness of snake species in the Atlantic Forest of Niterói and other regions of the state of Rio de Janeiro (Raw data available in Suppl. material 2).

**Figure 3.**
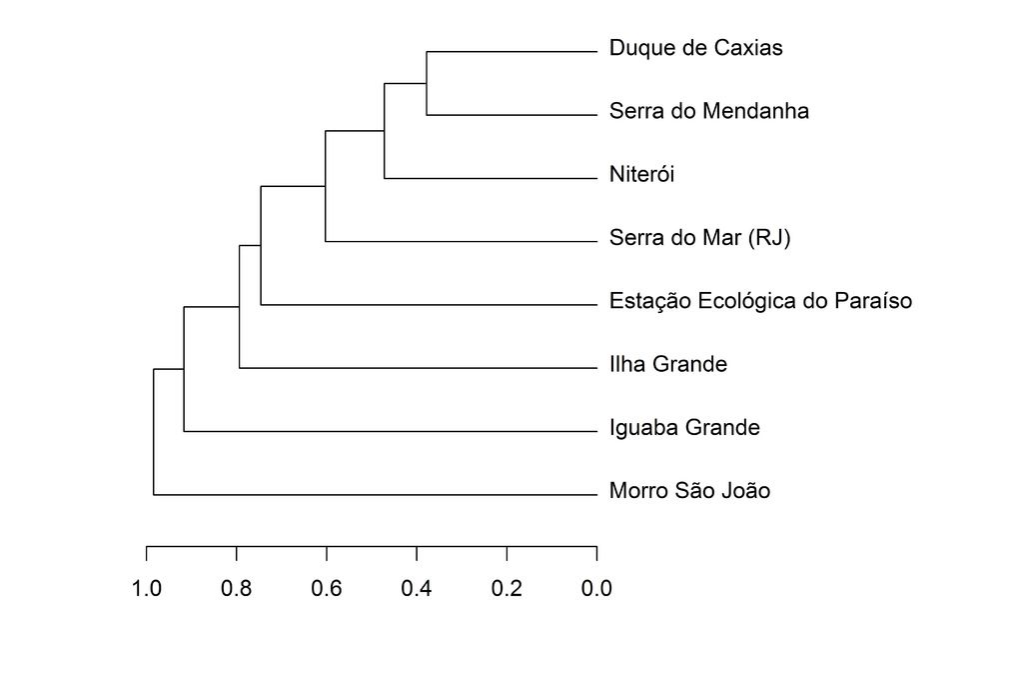
Dendrogram representing a cluster analysis, with Jaccard’s coefficient and centroid clustering method showing the taxonomic similarities between the Atlantic Forest of Niterói and Atlantic Forest in other municipalities of Rio de Janeiro (Raw data available in Suppl. material 2).

Most species are forest inhabitants (17 species, 61%), with a smaller percentage (39%) inhabiting both forested and open areas. All of them are typical of the Altantic Forest Domain. However, none of them exclusively inhabit open areas. The most species rich locality is the Serra do Mar with 60 species, followed by Duque de Caxias with 33 species (Fig. 2). We also recorded thirteen endemic Atlantic Forest species. The species composition is most similar to those of Duque de Caxias, Serra do Medanha and Estação Ecológica do Paraíso. However, the analysis did not recover distinct species clusters for the snakes in the Atlantic Forest of the state of Rio de Janeiro (see Fig. 3). All species recorded were listed as Least Concern (LC) in the Brazilian National Redlist (ICMBio 2014) since they are common in the Atlantic Forest and have a wide distribution range (see Checklist).

## Checklists

### List of species of snakes recorded in the municipality of Niterói, state of Rio de Janeiro, Brazil in this study

#### Liotyphlops wilderi

(Garman, 1883)

##### Ecological interactions

###### Conservation status

Least Concern

##### Distribution

Recorded in forested and open areas of the Atlantic Forest. Municipality of Niterói. State of Rio de Janeiro. Brazil

##### Notes

**Figure 4.**
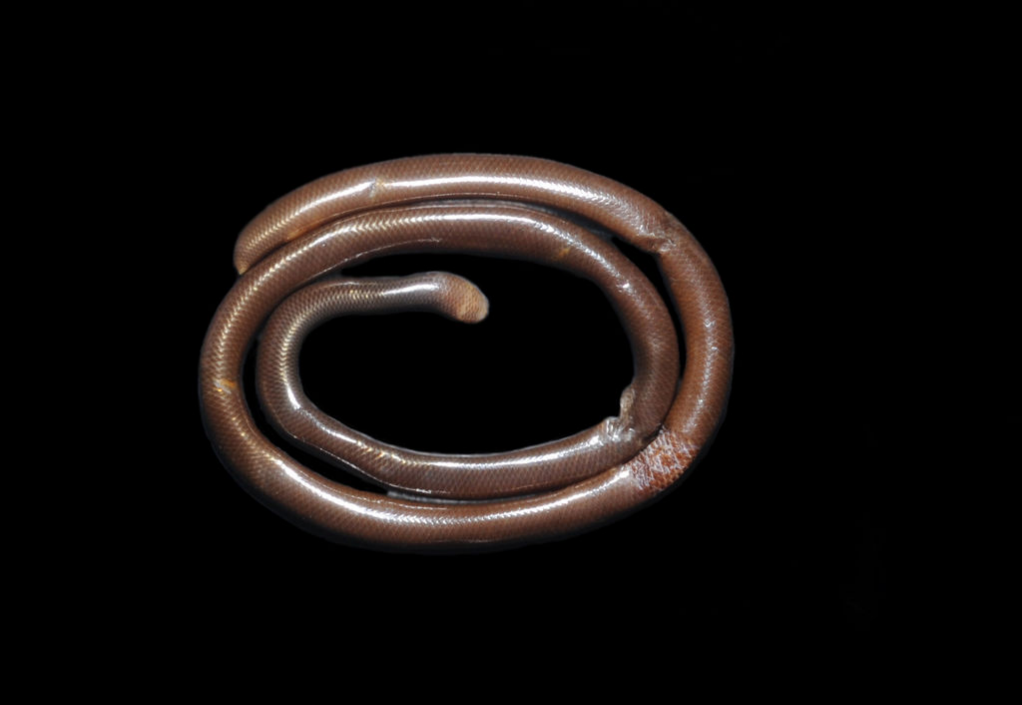
*Liotyphlops wilderi* (Anomalepididae) from Niterói, state of Rio de Janeiro, Brazil.

Endemic of the Atlantic Forest (Fig. 4). It is fossorial, nocturnal and diurnal, and feeds on arthropods.

#### Amerotyphlops brongersmianus

(Vanzolini, 1976)

##### Ecological interactions

###### Conservation status

Least Concern

##### Distribution

Recorded in forested areas of the Atlantic Forest. Municipality of Niterói. State of Rio de Janeiro. Brazil

##### Notes

**Figure 5.**
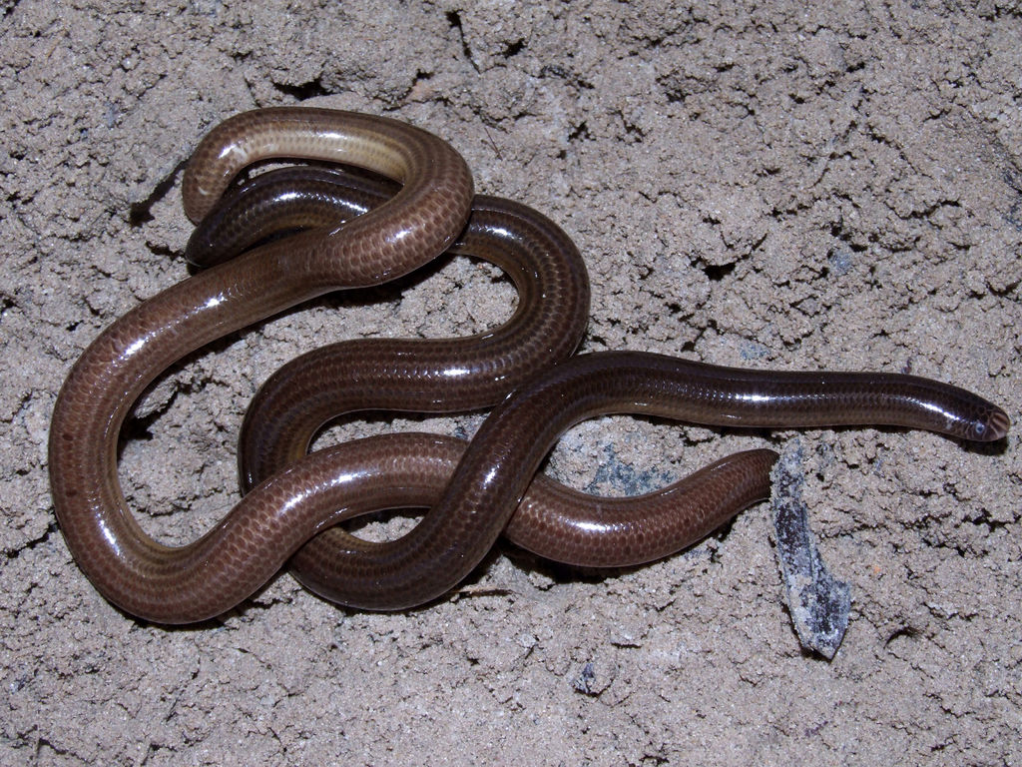
*Amerotyphlops brongersmianus* (Typhlopidae).

 It is fossorial, nocturnal and diurnal, and feeds on arthropods (Fig. 5).

#### Boa constrictor

Linnaeus, 1758

##### Ecological interactions

###### Conservation status

Least Concern

##### Distribution

Recorded in forested and open areas of the Atlantic Forest. Municipality of Niterói. State of Rio de Janeiro. Brazil

##### Notes

**Figure 6.**
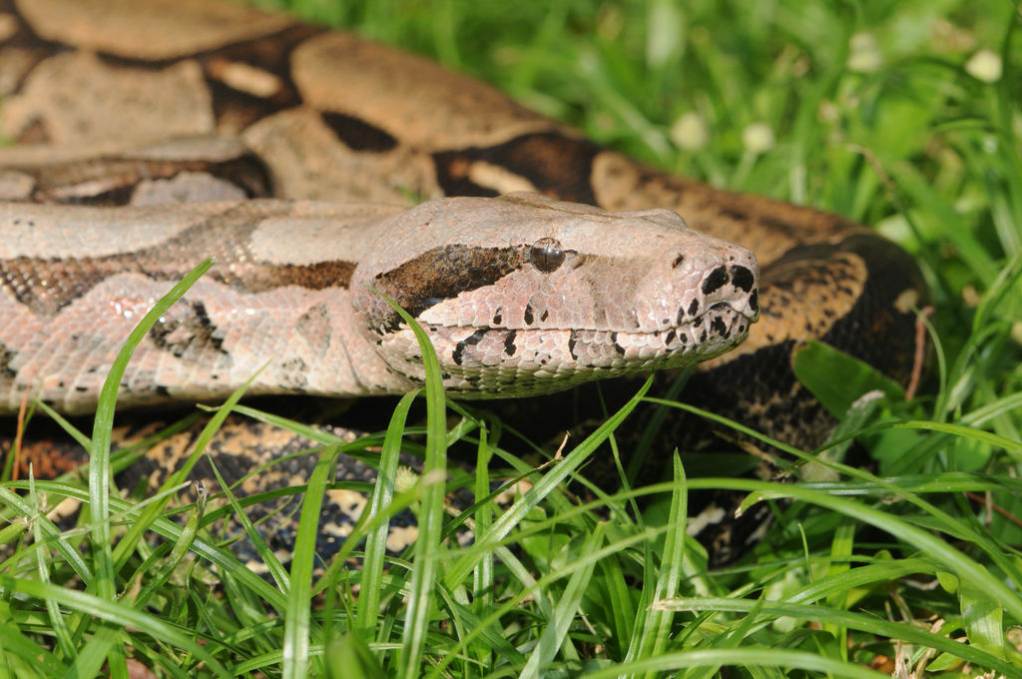
*Boa constrictor* (Boidae) from Niterói, state of Rio de Janeiro, Brazil.

It is semi-arboreal, diurnal and nocturnal and feeds mostly on vertebrates (birds and mammals) (Fig. 6).

#### Corallus hortulanus

(Linnaeus, 1758)

##### Ecological interactions

###### Conservation status

Least Concern

##### Distribution

Recorded in forested areas of the Atlantic Forest. Municipality of Niterói. State of Rio de Janeiro. Brazil

##### Notes

**Figure 7.**
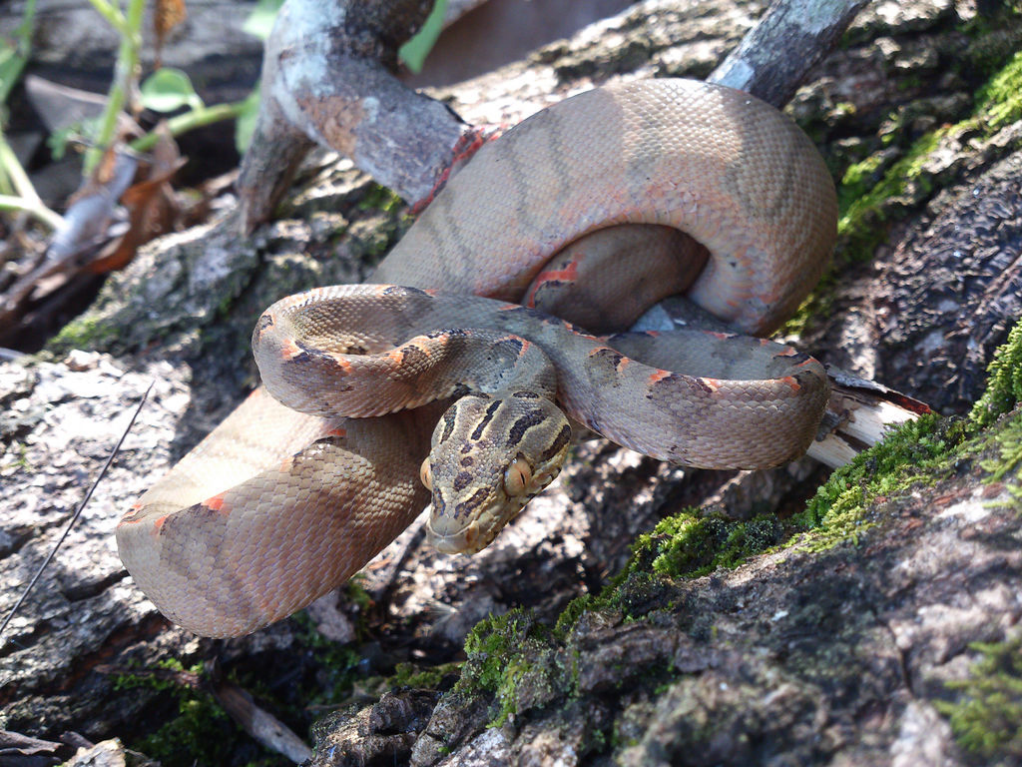
*Corallus hortulanus* (Boidae). Photo: G. Jones.

It is arboreal, nocturnal and feeds mostly on vertebrates (birds and mammals) (Fig. 7).

#### Chironius bicarinatus

(Wied, 1820)

##### Ecological interactions

###### Conservation status

Least Concern

##### Distribution

Recorded in forested areas of the Atlantic Forest. Municipality of Niterói. State of Rio de Janeiro. Brazil

##### Notes

**Figure 8.**
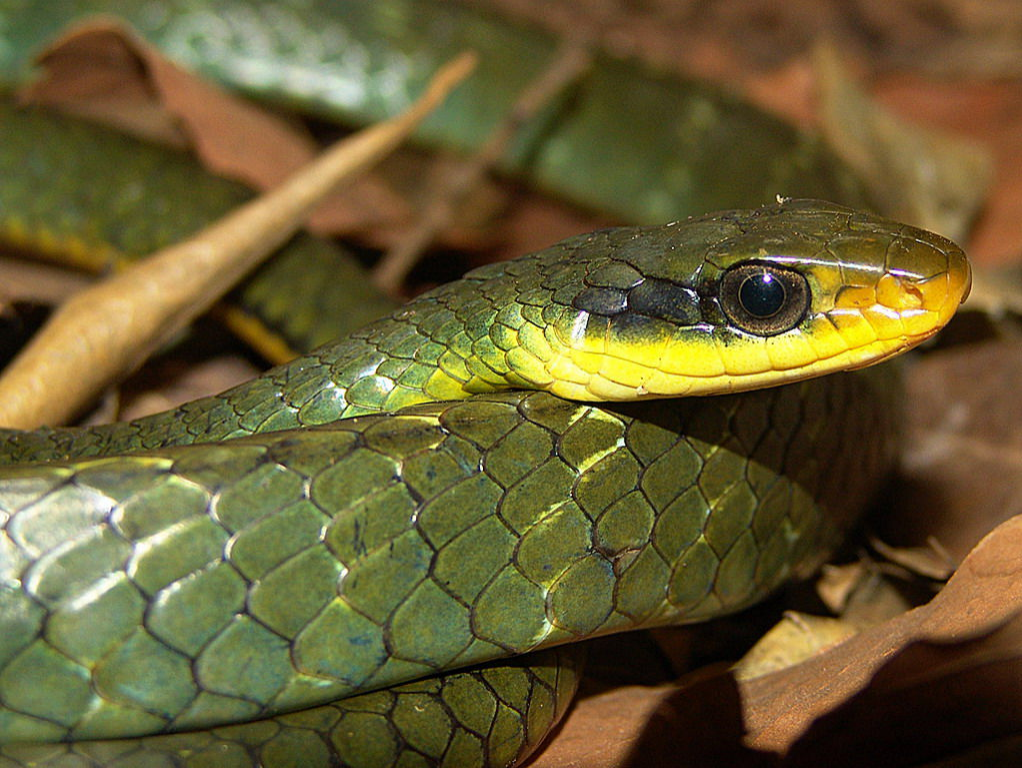
*Chironius bicarinatus* (Colubridae). Photo: D. Loebmann.

Endemic of the Atlantic Forest (Fig. 8). It is semi-arboreal, diurnal and feeds mostly on frogs.

#### Chironius exoletus

(Linnaeus, 1758)

##### Ecological interactions

###### Conservation status

Least Concern

##### Distribution

Recorded in forested areas of the Atlantic Forest. Municipality of Niterói. State of Rio de Janeiro. Brazil

##### Notes

**Figure 9.**
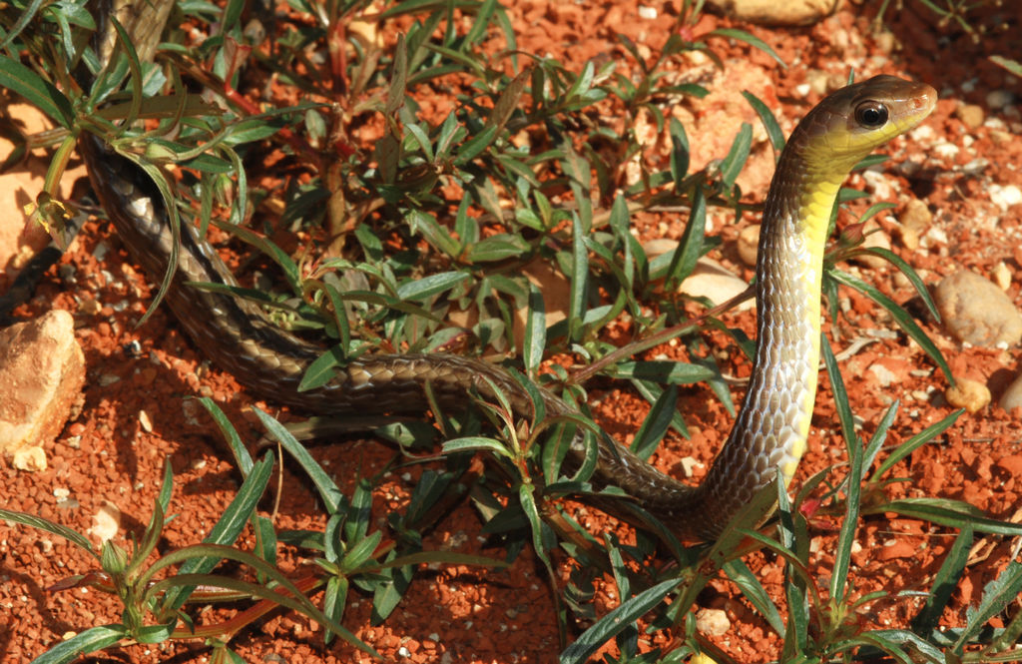
*Chironius exoletus* (Colubridae).

It is semi-arboreal, diurnal and feeds mostly on frogs (Fig. 9).

#### Chironius laevicollis

(Wied, 1824)

##### Ecological interactions

###### Conservation status

Least Concern

##### Distribution

Recorded in forested areas of the Atlantic Forest. Municipality of Niterói. State of Rio de Janeiro. Brazil

##### Notes

**Figure 10.**
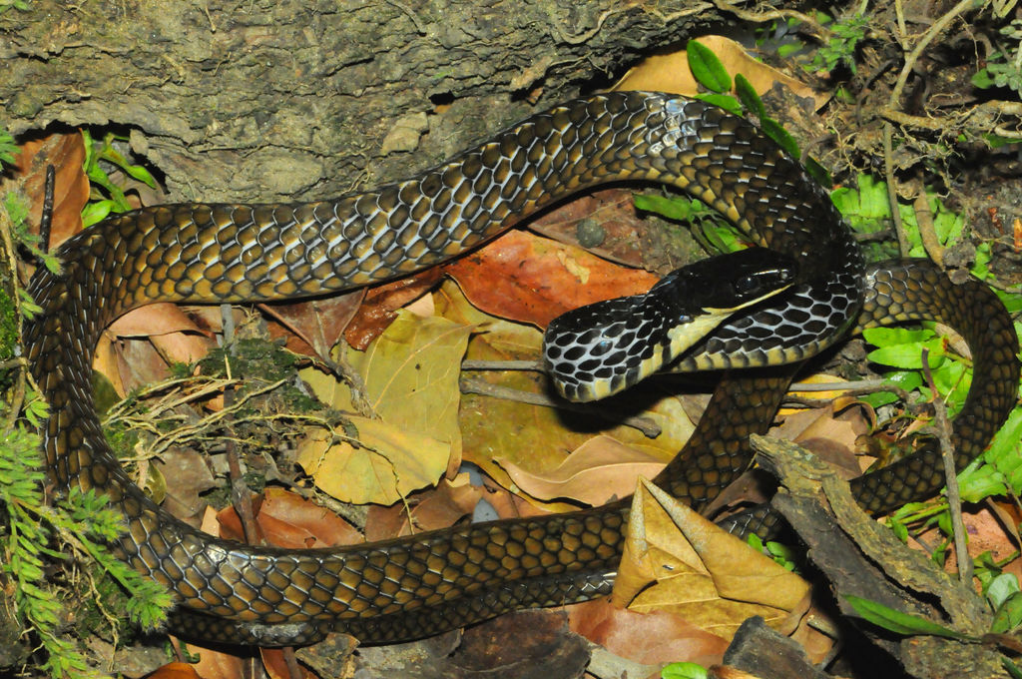
*Chironius laevicollis* (Colubridae) from Niterói, state of Rio de Janeiro, Brazil.

Endemic of the Atlantic Forest (Fig. 10). It is semi-arboreal, diurnal and feeds mostly on frogs.

#### Leptophis ahaetulla ahaetulla

(Wied, 1824)

##### Ecological interactions

###### Conservation status

Least Concern

##### Distribution

Recorded in forested and open areas of the Atlantic Forest. Municipality of Niterói. State of Rio de Janeiro. Brazil

##### Notes

**Figure 11.**
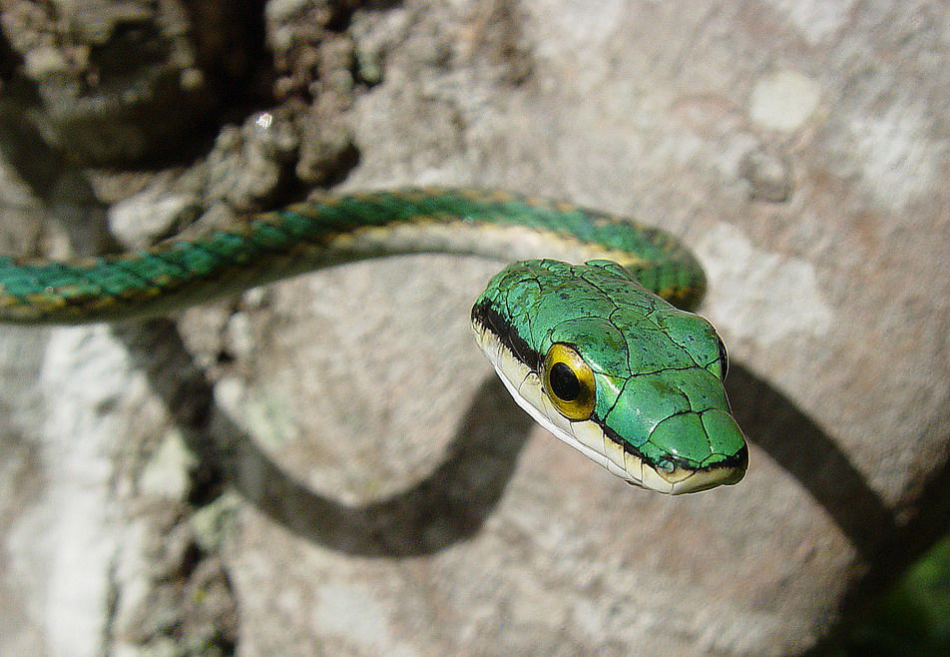
*Leptophis ahaetulla* (Colubridae). Photo: D. Loebmann.

It is semi-arboreal, diurnal and feeds on vertebrates (frogs, lizards and birds) (Fig. 11).

#### Mastigodryas bifossatus

(Raddi, 1820)

##### Ecological interactions

###### Conservation status

Least Concern

##### Distribution

Recorded in forested and open areas of the Atlantic Forest. Municipality of Niterói. State of Rio de Janeiro. Brazil

##### Notes

**Figure 12.**
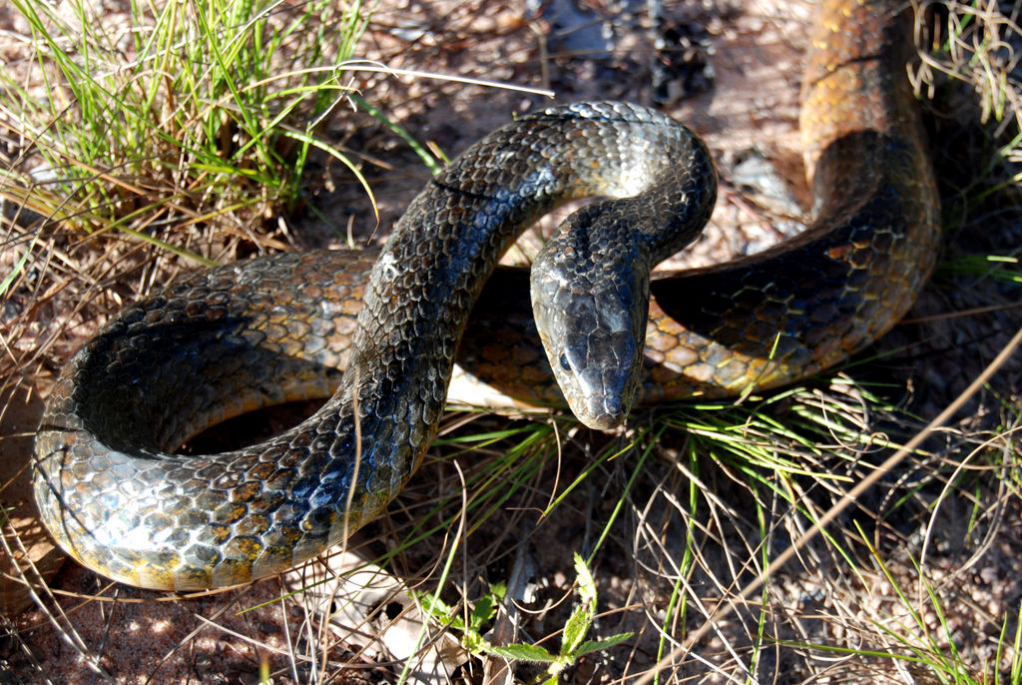
*Mastigodryas bifossatus* (Colubridae).

It is terrestrial, diurnal and feeds on vertebrates (frogs and mammals) (Fig. 12).

#### Spilotes pullatus anomalepis

(Linnaeus, 1758)

##### Ecological interactions

###### Conservation status

Least Concern

##### Distribution

Recorded in forested and open areas of the Atlantic Forest. Municipality of Niterói. State of Rio de Janeiro. Brazil

##### Notes

**Figure 13.**
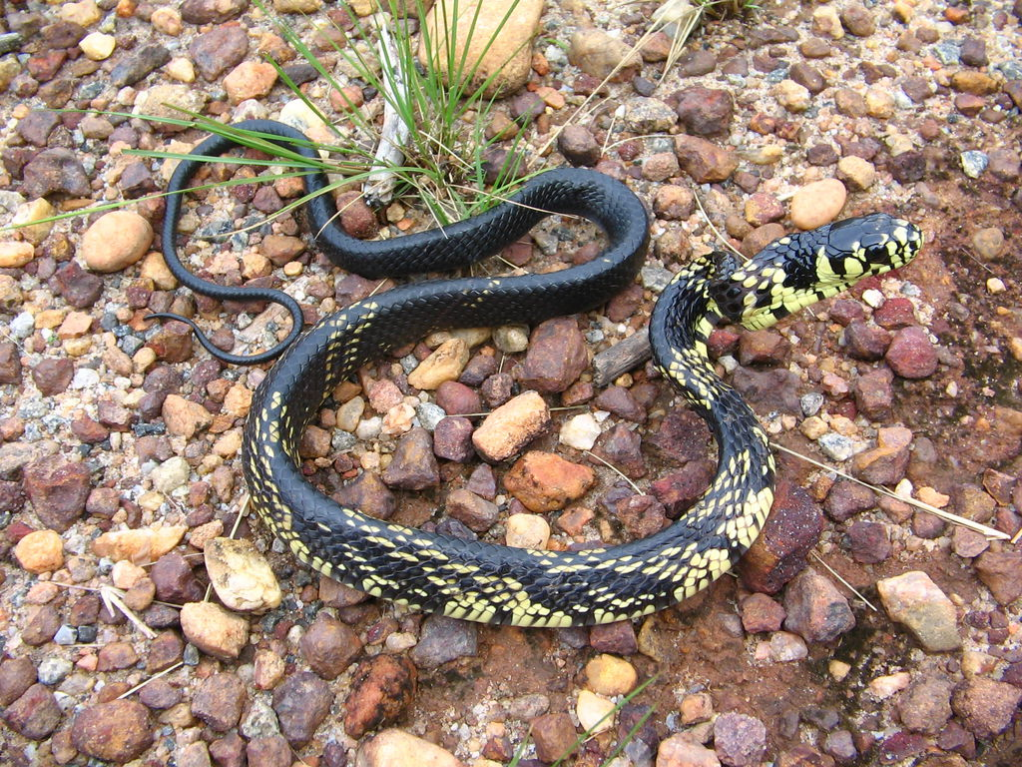
*Spilotes pullatus anomalepis* (Colubridae). Photo: W. Pessoa.

Endemic of the Atlantic Forest (Fig. 13). It is arboreal, diurnal and feeds on vertebrates (birds and mammals).

#### Spilotes sulphureus poecilostoma

(Wied, 1824)

##### Ecological interactions

###### Conservation status

Least Concern

##### Distribution

Recorded in forested areas of the Atlantic Forest. Municipality of Niterói. State of Rio de Janeiro. Brazil

##### Notes

**Figure 14.**
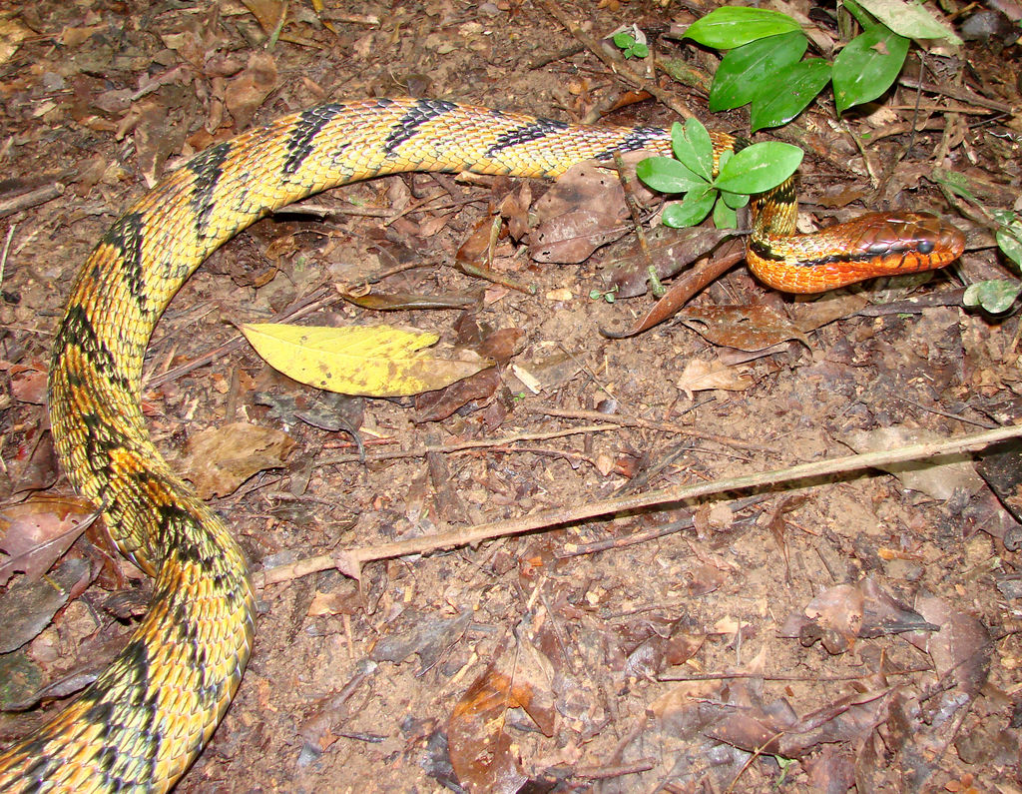
*Spilotes sulphureus poecilostoma* (Colubridae) from Niterói, state of Rio de Janeiro, Brazil. Photo: J. L. Pontes.

It is semi-arboreal, diurnal and feeds on vertebrates (frogs, lizards and mammals) (Fig. 14).

#### Oxybelis aeneus

(Wagler in Spix, 1824)

##### Ecological interactions

###### Conservation status

Least Concern

##### Distribution

Recorded in forested and open areas of the Atlantic Forest. Municipality of Niterói. State of Rio de Janeiro. Brazil

##### Notes

**Figure 15.**
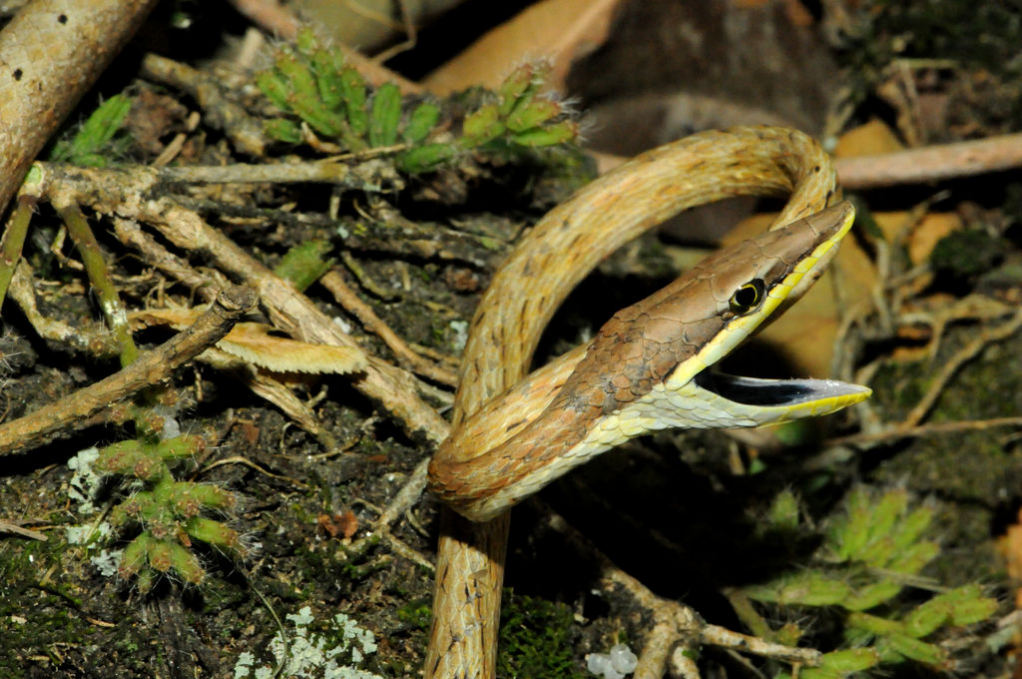
*Oxybelis aeneus* (Colubridae) from Niterói, state of Rio de Janeiro, Brazil.

It is arboreal, diurnal and feeds on vertebrates (lizards) (Fig. 15).

#### Clelia plumbea

(Wied, 1820)

##### Ecological interactions

###### Conservation status

Least Concern

##### Distribution

Recorded in forested areas of the Atlantic Forest. Municipality of Niterói. State of Rio de Janeiro. Brazil

##### Notes

**Figure 16.**
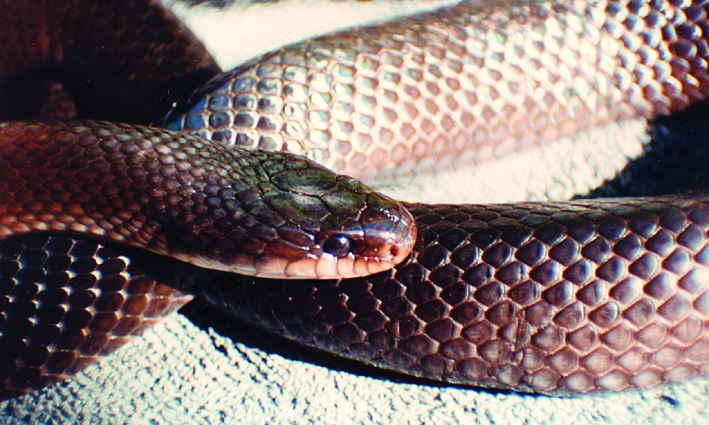
*Clelia plumbea* (Dipsadidae). Photo: M. A. Freitas.

It is terrestrial, nocturnal and feeds on vertebrates (snakes and lizards) (Fig. 16).

#### Elapomorphus quinquelineatus

(Raddi, 1820)

##### Ecological interactions

###### Conservation status

Least Concern

##### Distribution

Recorded in forested areas of the Atlantic Forest. Municipality of Niterói. State of Rio de Janeiro. Brazil

##### Notes

**Figure 17.**
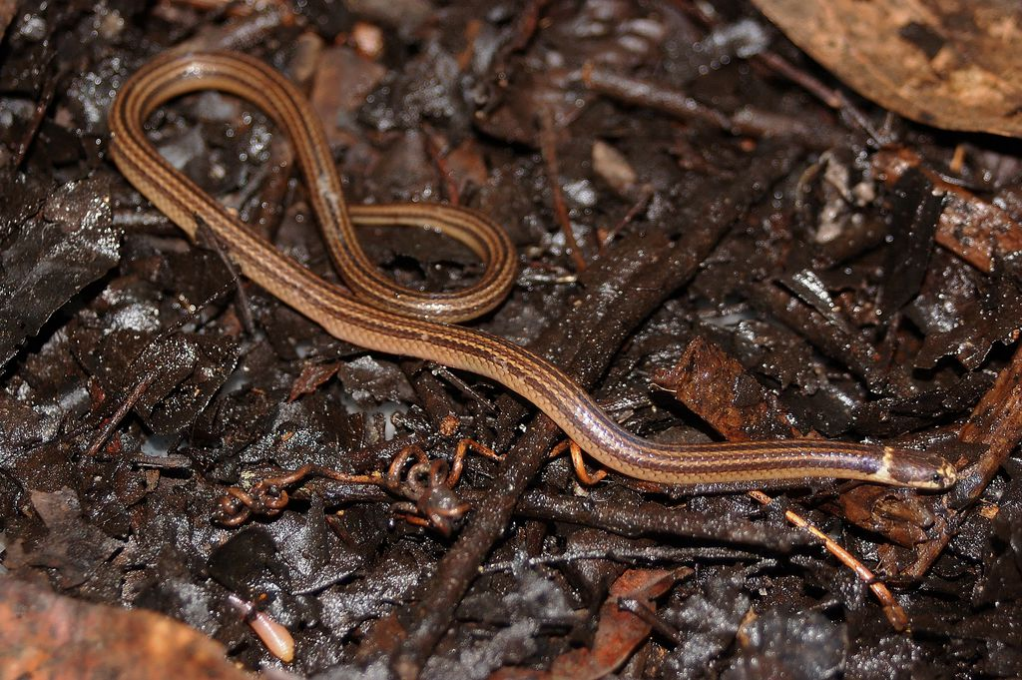
*Elapomorphus quinquelineatus* (Dipsadidae) from Niterói, state of Rio de Janeiro, Brazil. Photo: J. L. Pontes.

Endemic of the Atlantic Forest (Fig. 17). It is fossorial, diurnal and feeds on vertebrates (amphisbaena and snakes).

#### Erythrolamprus aesculapii

(Linnaeus, 1766)

##### Ecological interactions

###### Conservation status

Least Concern

##### Distribution

Recorded in forested areas of the Atlantic Forest. Municipality of Niterói. State of Rio de Janeiro. Brazil

##### Notes

**Figure 18.**
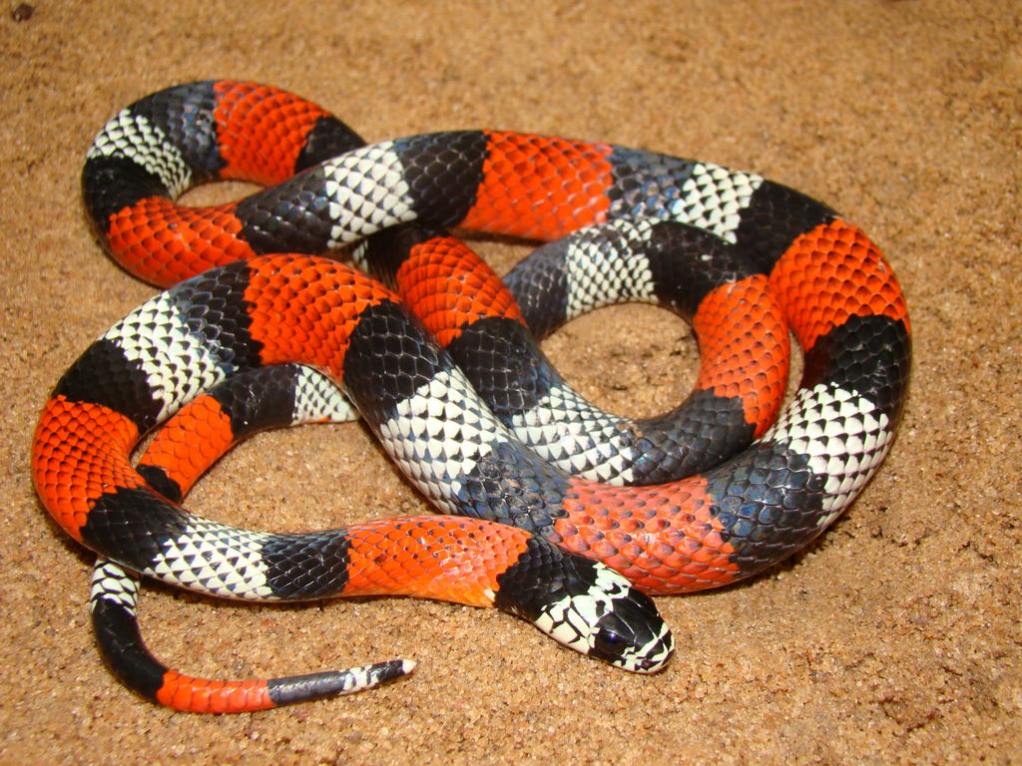
*Erythrolamprus aesculapii* (Dipsadidae). Photo: M. A. Freitas.

It is terrestrial, diurnal and feeds on vertebrates (amphisbaena, snakes and eventually lizards) (Fig. 18)

#### Erythrolamprus miliaris orinus

(Linnaeus, 1758)

##### Ecological interactions

###### Conservation status

Least Concern

##### Distribution

Recorded in forested areas of the Atlantic Forest. Municipality of Niterói. State of Rio de Janeiro. Brazil

##### Notes

**Figure 19.**
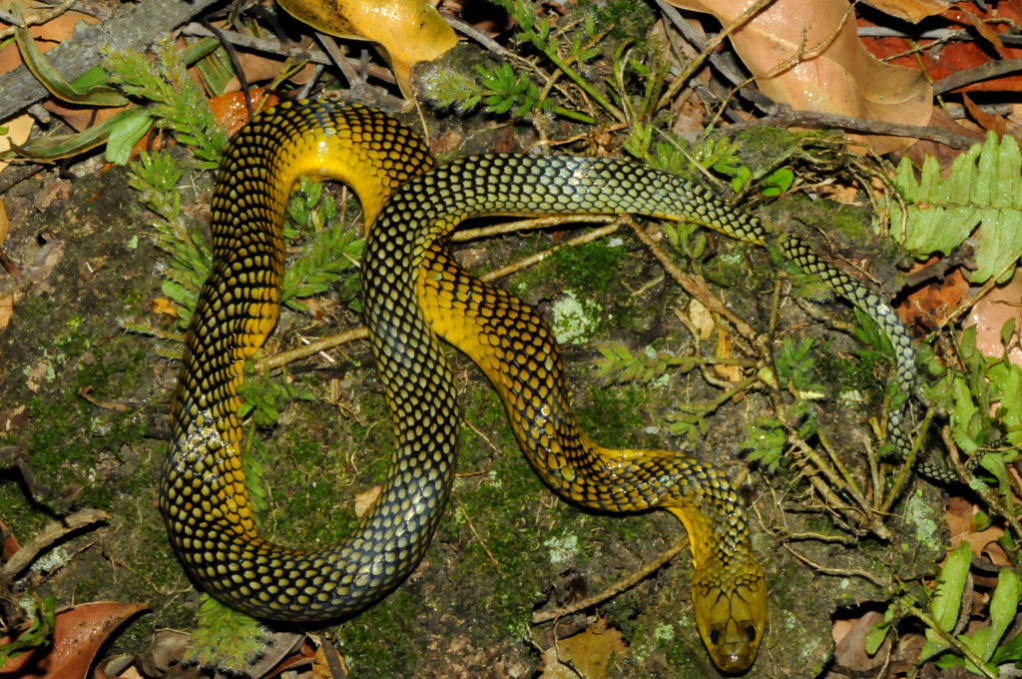
*Erythrolamprus miliaris orinus* (Dipsadidae) from Niterói, state of Rio de Janeiro, Brazil.

Endemic of the Atlantic Forest (Fig. 19). It is semi-aquatical, diurnal and feeds on vertebrates (frogs and fishes).

#### Erythrolamprus poecilogyrus poecilogyrus

(Wied, 1825)

##### Ecological interactions

###### Conservation status

Least Concern

##### Distribution

Recorded in forested and open areas of the Atlantic Forest. Municipality of Niterói. State of Rio de Janeiro. Brazil

##### Notes

**Figure 20.**
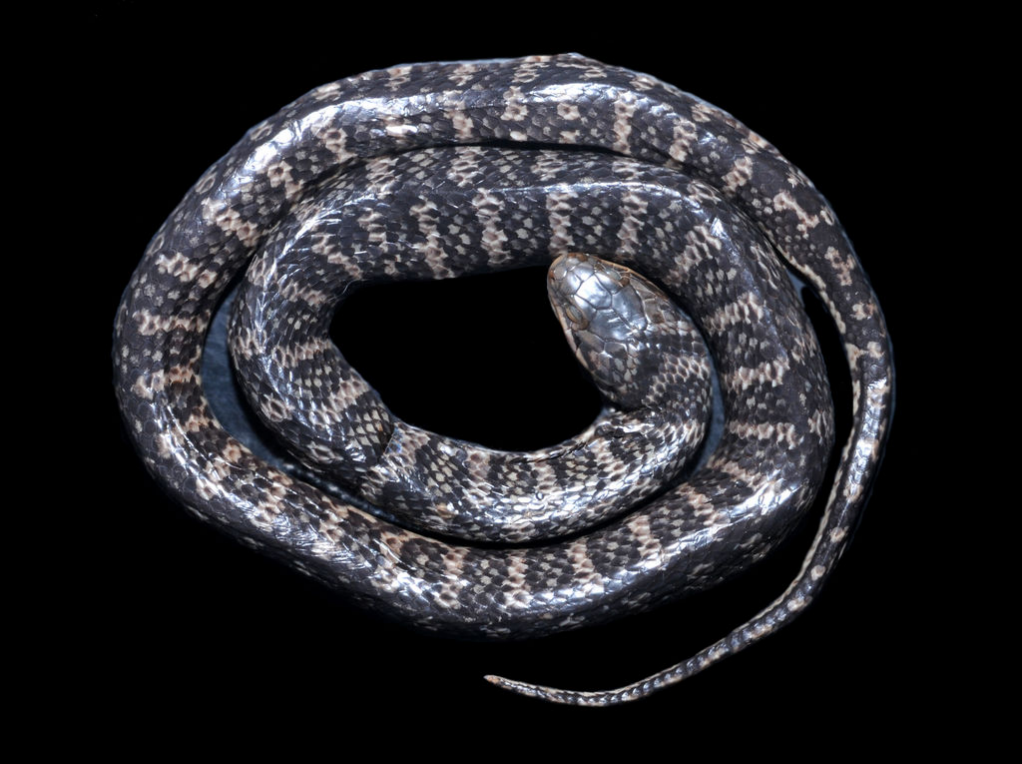
*Erythrolamprus poecilogyrus poecilogyrus* (Dipsadidae) from Niterói, state of Rio de Janeiro, Brazil.

Endemic of the Atlantic Forest (Fig. 20). It is semi-aquatical, diurnal and feeds on vertebrates (frogs and fishes).

#### Erythrolamprus poecilogyrus schotti

(Wied, 1825)

##### Ecological interactions

###### Conservation status

Least Concern

##### Distribution

Recorded in forested and open areas of the Atlantic Forest. Municipality of Niterói. State of Rio de Janeiro. Brazil

##### Notes

**Figure 21.**
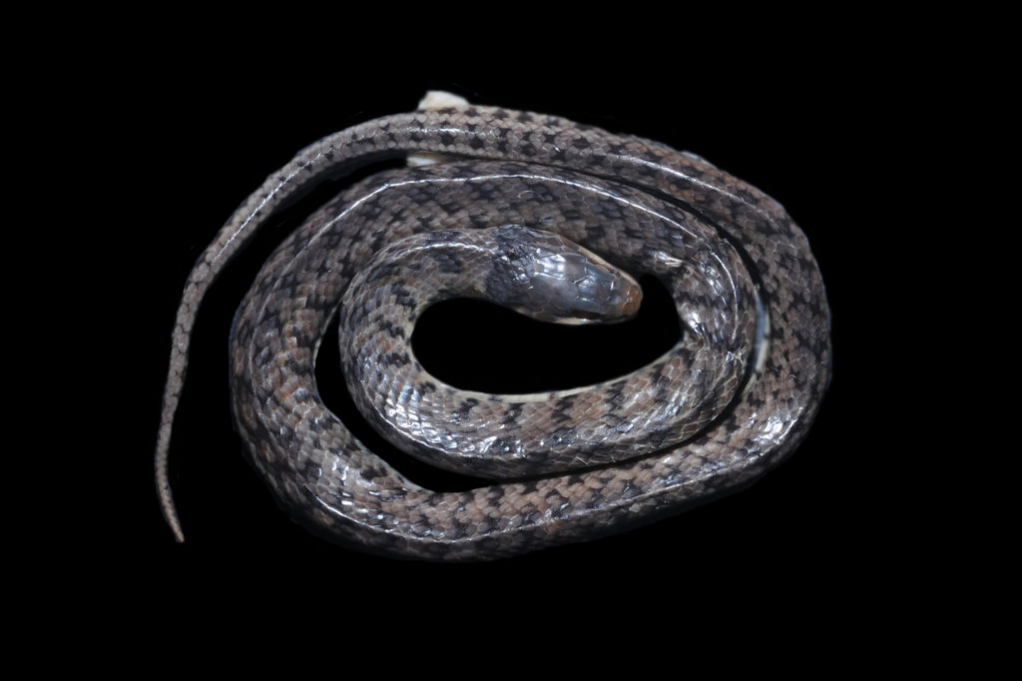
*Erythrolamprus poecilogyrus schotti* (Dipsadidae) from Niterói, state of Rio de Janeiro, Brazil.

It is terrestrial, diurnal and feeds on vertebrates (frogs).(Fig. 21)

#### Helicops carinicaudus

(Wied, 1825)

##### Ecological interactions

###### Conservation status

Least Concern

##### Distribution

Recorded in forested areas of the Atlantic Forest. Municipality of Niterói. State of Rio de Janeiro. Brazil

##### Notes

**Figure 22.**
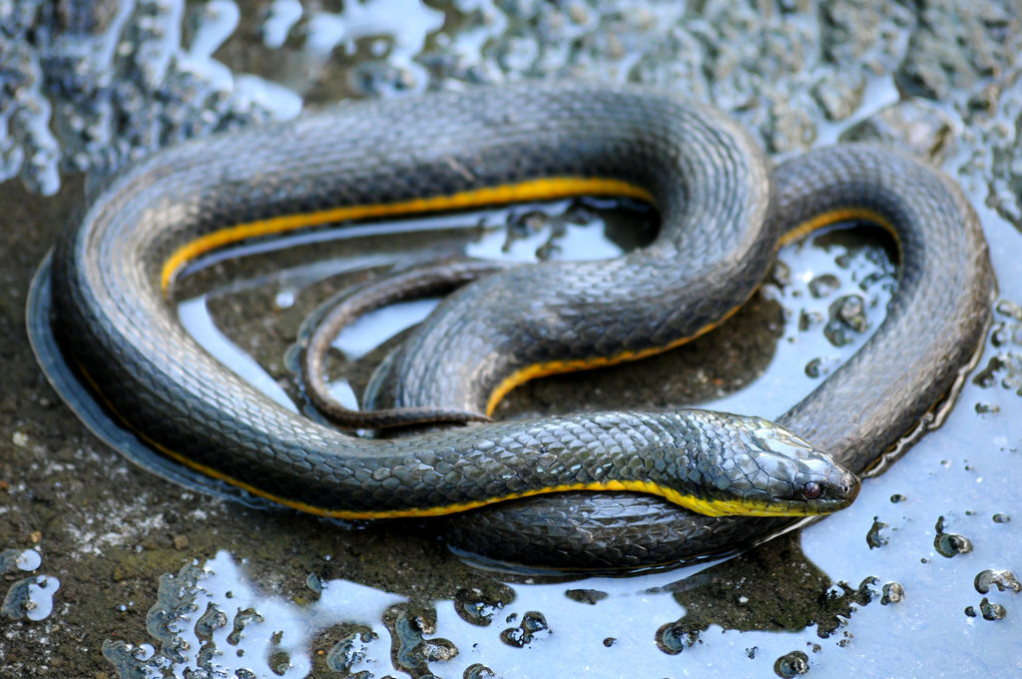
*Helicops carinicaudus* (Dipsadidae) from Niterói, state of Rio de Janeiro, Brazil.

It is aquatical, diurnal and feeds on vertebrates (fishes and frogs) (Fig. 22)

#### Oxyrhopus petolarius digitalis

(Reuss, 1834)

##### Ecological interactions

###### Conservation status

Least Concern

##### Distribution

Recorded in forested and open areas of the Atlantic Forest. Municipality of Niterói. State of Rio de Janeiro. Brazil

##### Notes

**Figure 23.**
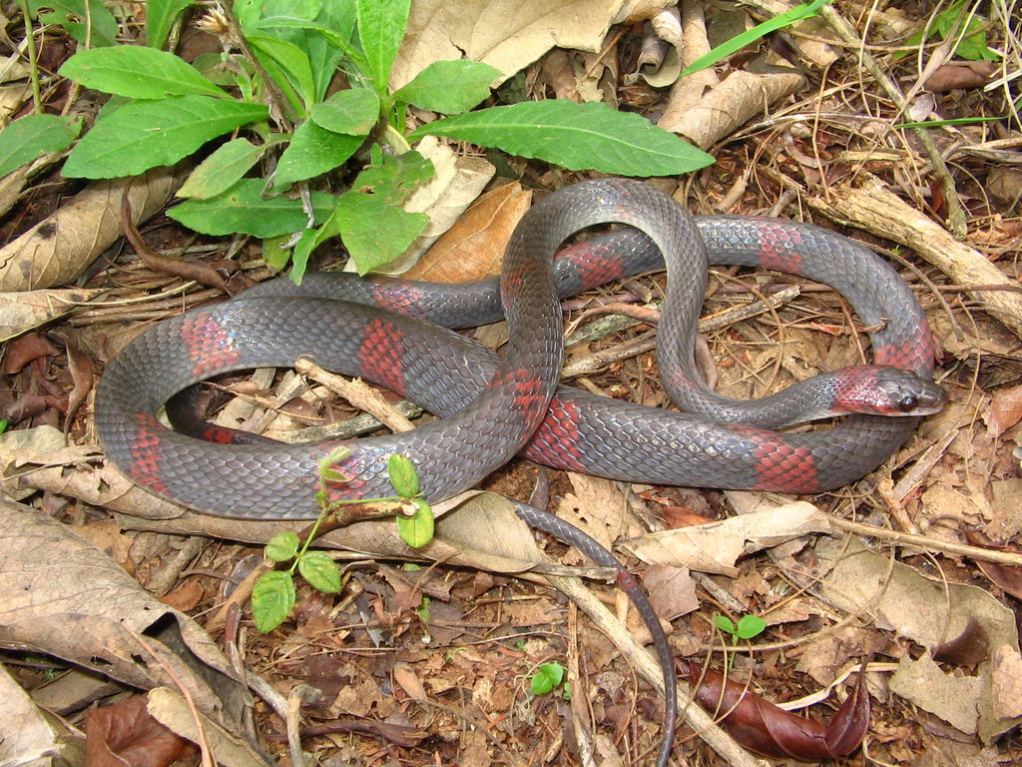
*Oxyrhopus petolarius digitalis* (Dipsadidae). Photo: M. A. Freitas

It is terrestrial, nocturnal and feeds on vertebrates (lizards and mammals) (Fig. 23)

#### Oxyrhopus clathratus

Duméril, Bibron & Duméril, 1854

##### Ecological interactions

###### Conservation status

Least Concern

##### Distribution

Recorded in forested and open areas of the Atlantic Forest. Municipality of Niterói. State of Rio de Janeiro. Brazil

##### Notes

**Figure 24.**
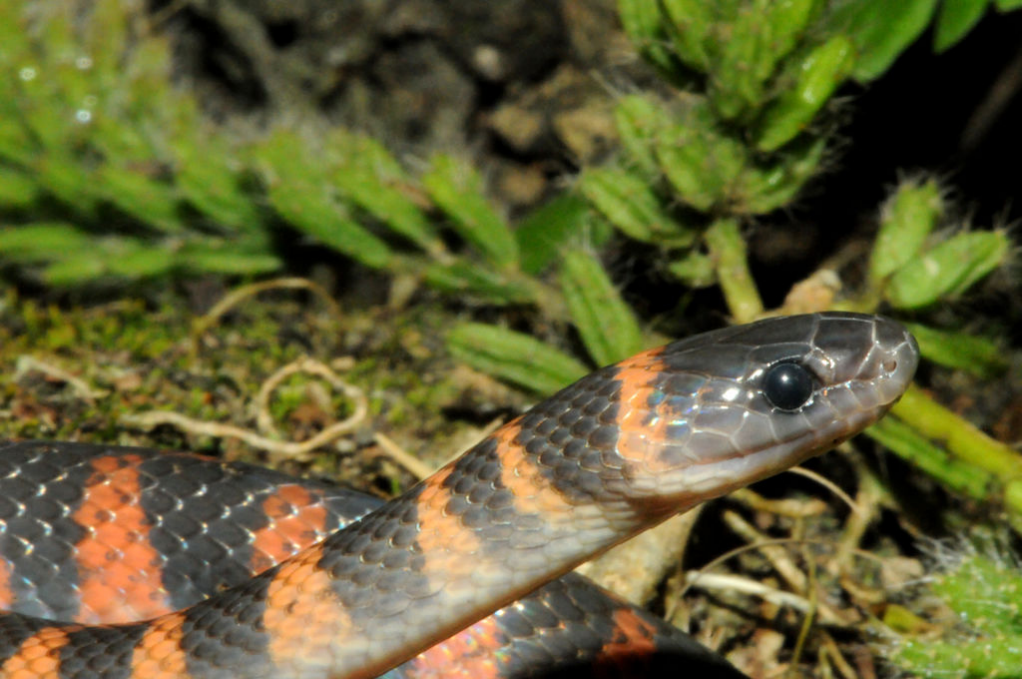
*Oxyrhopus clathratus* (Dipsadidae) from Niterói, state of Rio de Janeiro, Brazil.

Endemic of the Atlantic Forest (Fig. 24). It is terrestrial, nocturnal and feeds on vertebrates (lizards and mammals).

#### Philodryas olfersii olfersii

(Liechtenstein, 1823)

##### Ecological interactions

###### Conservation status

Least Concern

##### Distribution

Recorded in forested and open areas of the Atlantic Forest. Municipality of Niterói. State of Rio de Janeiro. Brazil

##### Notes

**Figure 25.**
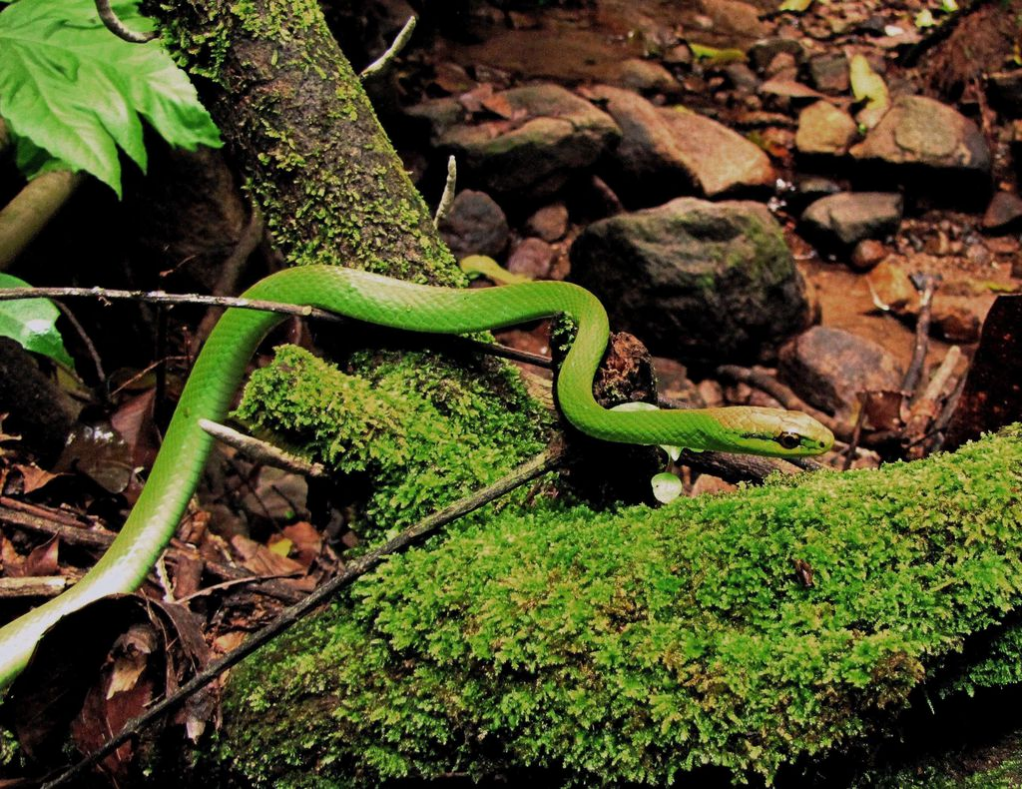
*Philodryas olfersii olfersii* (Dipsadidae). Photo: J. L. Pontes.

It is semi-arboreal, diurnal and feeds on vertebrates (lizards, mammals and birds) (Fig. 25)

#### Philodryas patagoniensis

(Girard, 1858)

##### Ecological interactions

###### Conservation status

Least Concern

##### Distribution

Recorded in forested and open areas of the Atlantic Forest. Municipality of Niterói. State of Rio de Janeiro. Brazil

##### Notes

**Figure 26.**
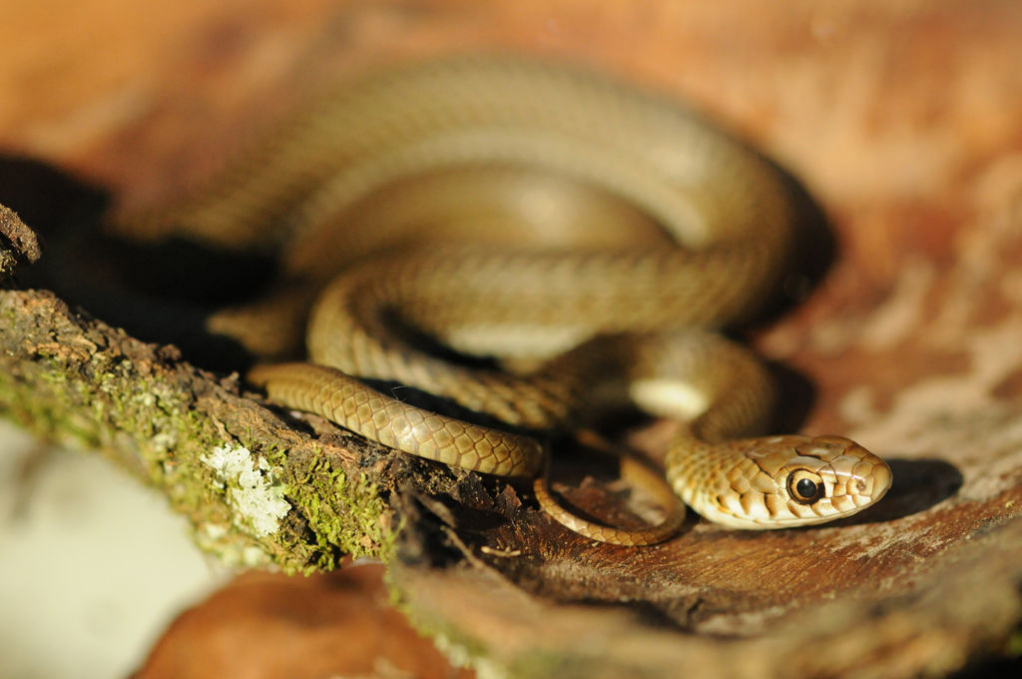
*Philodryas patagoniensis* (Dipsadidae) from Niterói, state of Rio de Janeiro, Brazil.

It is terrestrial, diurnal and feeds on vertebrates (lizards and mammals) (Fig. 26)

#### Sibynomorphus neuwiedi

(Ihering, 1911)

##### Ecological interactions

###### Conservation status

Least Concern

##### Distribution

Recorded in forested areas of the Atlantic Forest. Municipality of Niterói. State of Rio de Janeiro. Brazil

##### Notes

**Figure 27.**
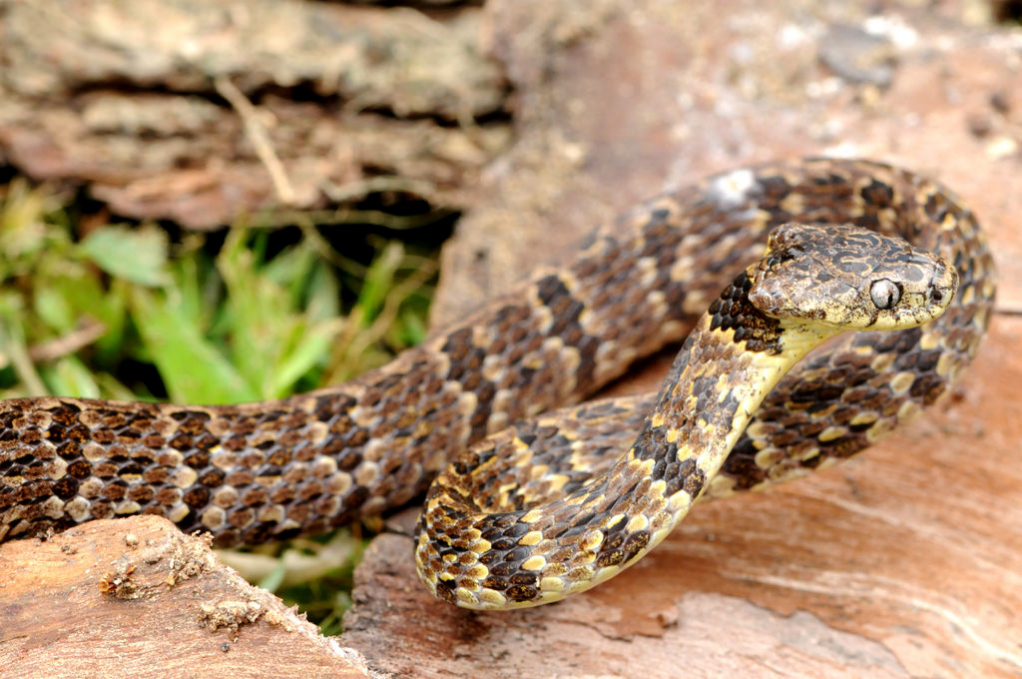
*Sibynomorphus neuwiedi* (Dipsadidae) from Niterói, state of Rio de Janeiro, Brazil.

Endemic of the Atlantic Forest (Fig. 27). It is terrestrial, nocturnal and feeds on molluscs.

#### Thamnodynastes cf. nattereri

(Mikan, 1828)

##### Ecological interactions

###### Conservation status

Least Concern

##### Distribution

Recorded in forested areas of the Atlantic Forest. Municipality of Niterói. State of Rio de Janeiro. Brazil

##### Notes

**Figure 28.**
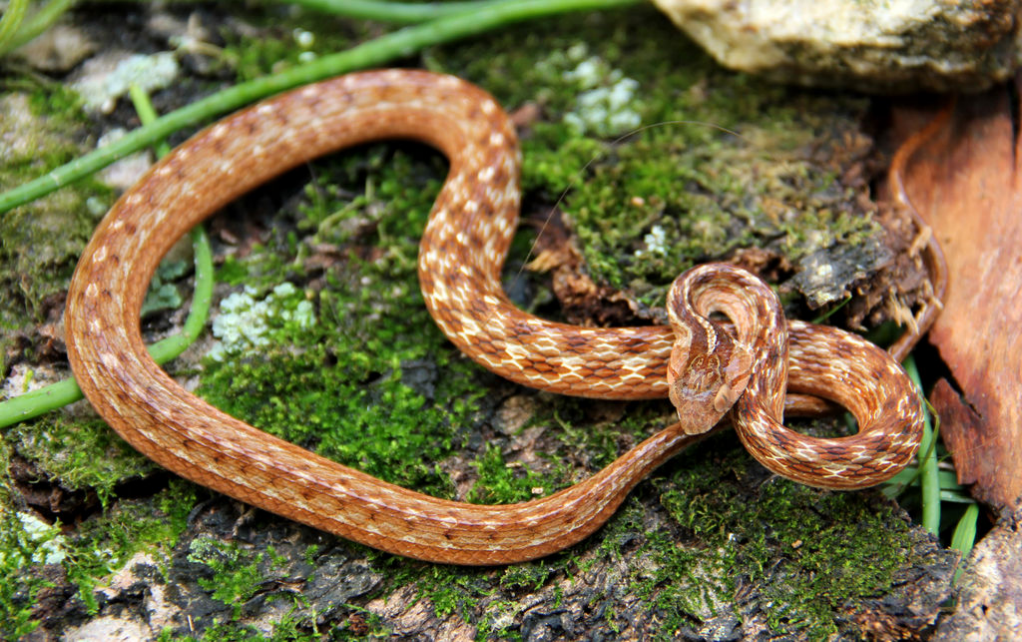
Thamnodynastes cf. nattereri (Dipsadidae) from Niterói, state of Rio de Janeiro, Brazil.

It is semi-arboreal, nocturnal and feeds on vertebrates (frogs) (Fig. 28). See taxonomic notes for the species taxonomic status.

#### Xenodon neuwiedii

Günther, 1863

##### Ecological interactions

###### Conservation status

Least Concern

##### Distribution

Recorded in forested and open areas of the Atlantic Forest. Municipality of Niterói. State of Rio de Janeiro. Brazil

##### Notes

**Figure 29.**
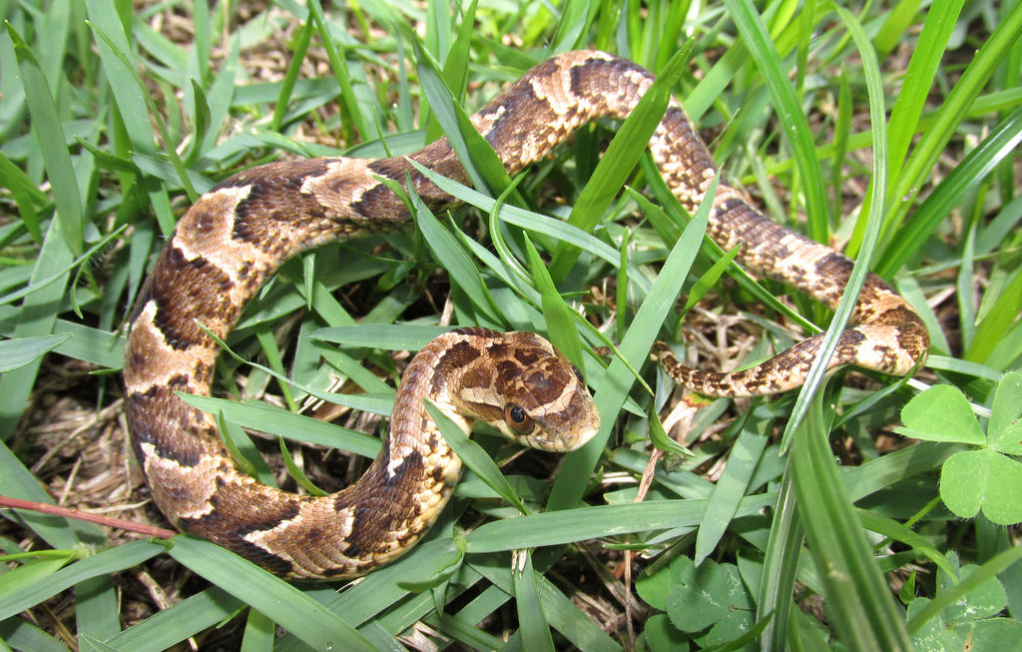
*Xenodon neuwiedii* (Dipsadidae) from Niterói, state of Rio de Janeiro, Brazil.

It is terrestrial, diurnal and feeds on vertebrates (frogs) (Fig. 29)

#### Micrurus corallinus

(Merrem, 1820)

##### Ecological interactions

###### Conservation status

Least Concern

##### Distribution

Recorded in forested areas of the Atlantic Forest. Municipality of Niterói. State of Rio de Janeiro. Brazil

##### Notes

**Figure 30.**
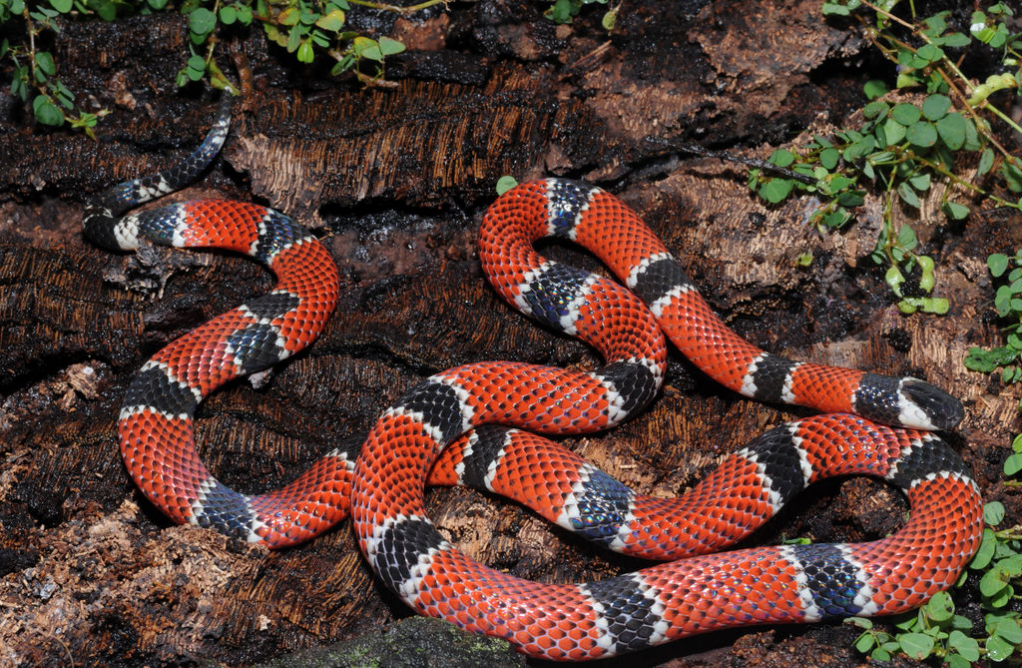
*Micrurus corallinus* (Elapidae) from Niterói, state of Rio de Janeiro, Brazil.

Endemic of the Atlantic Forest (Fig. 30). It is fossorial, diurnal and nocturnal and feeds on vertebrates (lizards and snakes).

#### Bothrops jararaca

(Wied, 1824)

##### Ecological interactions

###### Conservation status

Least Concern

##### Distribution

Recorded in forested areas of the Atlantic Forest. Municipality of Niterói. State of Rio de Janeiro. Brazil

##### Notes

**Figure 31.**
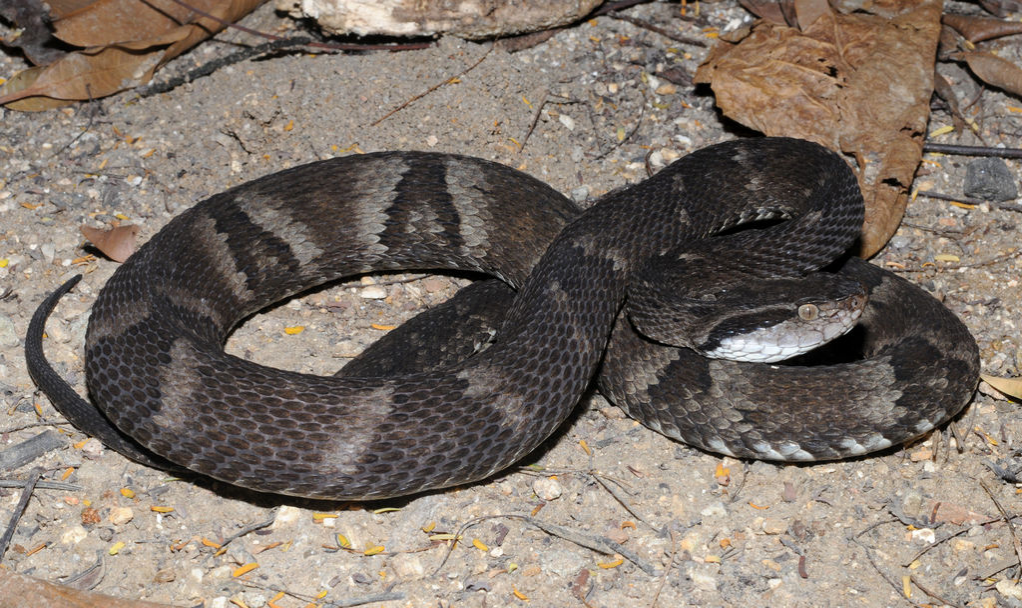
*Bothrops jararaca* (Viperidae) from Niterói, state of Rio de Janeiro, Brazil.

Endemic of the Atlantic Forest (Fig. 31). It is terrestrial, nocturnal and feeds on vertebrates (mammals, frogs and lizards).

#### Bothrops jararacussu

Lacerda, 1884

##### Ecological interactions

###### Conservation status

Least Concern

##### Distribution

Recorded in forested areas of the Atlantic Forest. Municipality of Niterói. State of Rio de Janeiro. Brazil

##### Notes

**Figure 32.**
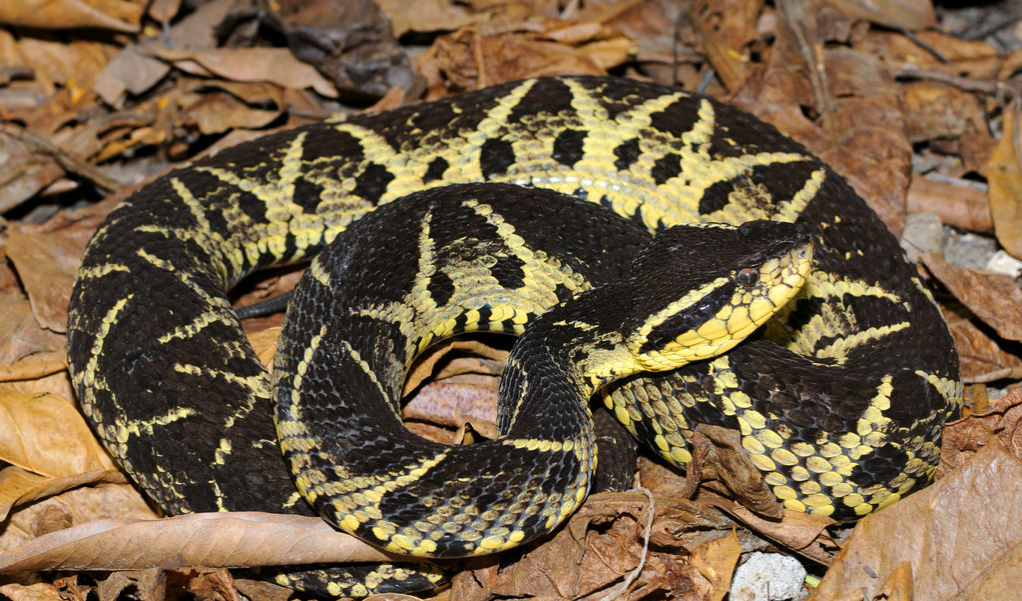
*Bothrops jararacussu* (Viperidae) from Niterói, state of Rio de Janeiro, Brazil.

 It is terrestrial, nocturnal and feeds on vertebrates (mammals and frogs) (Fig. 32).

## Discussion

The species richness found in Niterói represents 34% of the snake species known for the state of Rio de Janeiro (Rocha et al. 2004) and 7.3% of the species known for Brazil (Costa and Bérnils 2014). Additionally, the richness recorded corresponds to 47% of the snake species occurring in the southern portion of the Atlantic Forest (Marques et al. 2004). The richness found in Niterói is similar to that reported for other localities in the Atlantic Forest, such as Viçosa (Minas Gerais; 27 species; Costa et al. 2010), Duque de Caxias (Rio de Janeiro, 33 spp., Salles and Silva-Soares 2010), Serra do Medanha (Rio de Janeiro, 27 spp., Pontes and Rocha 2008) and Vitória (Espírito Santo, 27 spp., Silva-Soares et al. 2011). The surveys in protected areas had lower richness (e.g., Martins et al. 2012) when compared with our study. This is probably an artifact of the size of the area or might be a consequence of the environmental education program developed by the Vital Brazil Institute that receive venomous snakes species from the population for snakebite serum production (it also justify the higher abundance of *Bothrops jararaca* and *Micrurus corallinus* in our samples).

Information about diversity, taxonomy, and geographical distribution of species from scientific collections can be used for a broad range of purposes (Graham et al. 2004), including conservation. Thus, Brazilian scientific collections play a key role in recording part of the country’s biodiversity. For example, by analyzing specimens housed in scientific collections we were able to recover the snake richness of an area of Atlantic Forest that is today primarily covered by a large city.

Currently, no complete snake species list for the entire Atlantic Forest, let alone a list of the potentially threatened endemic species but, based on the literature (e.g., Cunha and Nascimento 1993, Cunha and Nascimento 1978, Marques et al. 2004, Strussmann and Sazima 1993), it seems that 13 species are endemic to this biome (see checklist). Most of the species (17 species, 61%) are strictly forest inhabitants (e.g., species of *Chironius* and *Bothrops*), and eleven (39%) inhabit both open and forested areas (e.g., genus *Philodryas*; Argôlo 2004, Hartmann et al. 2009, Marques et al. 2009, Costa et al. 2010, Gaiarsa et al. 2013). Our results suggest that the Atlantic Forest remnants in Niterói are important for maintaining the high snake diversity, since most species are not adapted to live in urban environments (Marques et al. 2009).

The snake fauna of Niterói is similar to the nearby localities of Duque de Caxias, Serra do Medanha, and Estação Ecológica do Paraíso. This suggests that there is little turnover in snake species composition between lowland Atlantic Forest areas of the state of Rio de Janeiro (Salles and Silva-Soares 2010). It seems likely that the areas surrounding the Bay of Guanabara share the same species, and perhaps the same evolutionary history.

None of the species recorded were listed as threatened (ICMBio 2014). However, most of them are strongly associated with forested areas and are not adapted to live in small fragments, forest edges, or urban areas (Argôlo 2004, Marques et al. 2009). The Brazilian national redlist takes into account the whole species range and the occurrence of each species in protected areas. But the conservation status in each region can be different (e.g., Bressan et al. 2009).

Most of the protected areas in Brazil have less than 500 ha, an inadequate size to maintain their biota (Viana and Pinheiro 1998). Thus, strategies for biodiversity conservation should expand the boundaries of protected areas as part of the large potential area to be protected (Viana and Pinheiro 1998). The municipal and state protected areas are apparently effective in preserving this high snake richness in the study region (Viana and Pinheiro 1998). However, increased urbanization rate and population put the survival of species at risk and may considerably alter patterns of species richness and composition (e.g., Viviani et al. 2010). There is an increasing trend towards deforestation in Niterói due to the construction of ports and houses around the forest fragments in the Bay of Guanabara. This removal of vegetation cover could exert considerable pressure on these Atlantic Forest remnants over time. Thus, unprotected forested areas in and around Niterói (e.g., Maricá, São Gonçalo Saquarema, Araruama, Rio Bonito, and Itaboraí) should be protected. Our study reinforces the importance of forested areas in Niterói for protecting species diversity. We suggest that public policies related to environmental education should be implemented, as well as the expansion of protected areas and the creation of new ones.

### Taxonomic notes

*Erythrolamprus poecilogyrus schotti* (IVB 1399, 2010) is widely distributed in the Caatinga and Cerrado, with dubious records in the Atlantic Forest of Rio de Janeiro (Dixon and Markezich 1992). The specimens analyzed here have diagnostic characteristics of this subspecies, namely: 19-19-15 of dorsal rows, 154-161 ventral scales, eight supralabials, nine infralabials, and 53-56 subcaudals. Therefore, we confirm that this subspecies occurs in Niterói.

The specimens of *Thamnodynastes* from Niterói have dorsal scales weakly keeled, ventral portion of the head immaculate, longitudinal stripes little conspicuous, posterior region of the vent darker, 3^rd^ and 4^th^ supralabials in contact with the orbits, 19 dorsal rows, loreal present, ventrals ranging from 141 to 165 in males and from 147 to 162 in females. These characteristics correspond to Thamnodynastes cf. nattereri (Franco and Ferreira 2002). The taxonomy of Thamnodynastes cf. nattereri is complicated, since the type material is lost (F. Franco *pers. comm*.). This species is morphologically similar to *T. hypoconia* that also occurs in the Atlantic Forest of Rio de Janeiro. However, it cannot be treated as *T. hypoconia* because the later has dorsal scales strongly keeled, anterior and posterior region of the vent homogeneous, with longitudinal stripes very conspicuous, and dark gular region (Franco and Ferreira 2002).

## Supplementary Material

Supplementary material 1 Snake richness from urban forest fragments of Niterói and surroundings, state of Rio de Janeiro, Southeastern Brazil Data type: Appendix 1
Brief description: Voucher numbers of specimens analyzed from the Municipality of Niterói housed in the following scientific collections: Coleção Científica Instituto Vital Brazil (IVB), Museu Nacional da Universidade Federal do Rio de Janeiro (MNRJ), and Museu de Zoologia da Universidade Estadual de Campinas (ZUEC).
File: oo_58178.docx Nathalie Citeli, Breno Hamdan, Thaís Barreto Guedes

Supplementary material 2 Raw data of occurrence of snakes in areas of the Atlantic Forest, Southeastern Brazil Data type: Binary matrix in Excel
Brief description: Binary matrix that was the base to run the cluster analysis with Jaccard’s coefficient and to draw the graphics provided in the results section (Fig. 31 and 32).
File: oo_63307.xlsx Nathalie Citeli, Breno Hamdan, Thaís Barreto Guedes

XML Treatment for Liotyphlops wilderi

XML Treatment for Amerotyphlops brongersmianus

XML Treatment for Boa constrictor

XML Treatment for Corallus hortulanus

XML Treatment for Chironius bicarinatus

XML Treatment for Chironius exoletus

XML Treatment for Chironius laevicollis

XML Treatment for Leptophis ahaetulla ahaetulla

XML Treatment for Mastigodryas bifossatus

XML Treatment for Spilotes pullatus anomalepis

XML Treatment for Spilotes sulphureus poecilostoma

XML Treatment for Oxybelis aeneus

XML Treatment for Clelia plumbea

XML Treatment for Elapomorphus quinquelineatus

XML Treatment for Erythrolamprus aesculapii

XML Treatment for Erythrolamprus miliaris orinus

XML Treatment for Erythrolamprus poecilogyrus poecilogyrus

XML Treatment for Erythrolamprus poecilogyrus schotti

XML Treatment for Helicops carinicaudus

XML Treatment for Oxyrhopus petolarius digitalis

XML Treatment for Oxyrhopus clathratus

XML Treatment for Philodryas olfersii olfersii

XML Treatment for Philodryas patagoniensis

XML Treatment for Sibynomorphus neuwiedi

XML Treatment for Thamnodynastes cf. nattereri

XML Treatment for Xenodon neuwiedii

XML Treatment for Micrurus corallinus

XML Treatment for Bothrops jararaca

XML Treatment for Bothrops jararacussu
