# Supplementary material for: Snake richness in urban forest fragments from Niterói and surroundings, state of Rio de Janeiro, southeastern Brazil
Source: Biodivers Data J. 2016 Feb 4;(4):e7145. doi: 10.3897/BDJ.4.e7145 (PMC4759410; doi:10.3897/BDJ.4.e7145)
Supplement: Supplementary material 1 — Snake richness from urban forest fragments of Niterói and surroundings, state of Rio de Janeiro, Southeastern Brazil [file biodiversity_data_journal-4-e7145-s001.docx]

**Appendix I**

Voucher numbers of specimens analyzed from the Municipality of Niterói housed in the the following scientific collections: Coleção Científica Instituto Vital Brazil (IVB), Museu Nacional da Universidade Federal do Rio de Janeiro (MNRJ), and Museu de Zoologia da Universidade Estadual de Campinas (ZUEC).

Anomalepididae: *Liotyphlops wilderi* (IVB 2358); Boidae: *Boa* *constrictor* (IVB 3381); *Corallus hortulanus* (IVB 1417, IVB 2404); Dipsadidae: *Erythrolamprus aesculapii* *aesculapii* (IVB 3090), *Erythrolamprus miliaris orinus* (IVB 594, 1098, 2324), *Erythrolamprus poecilogyrus poecilogyrus* (IVB 156, 609, 619, 634, 643, 786, 2008), *Erythrolamprus poecilogyrus schotti* (IVB 1399, 2010), *Elapomorphus quinquelineatus* (IVB 894)*, Clelia plumbea* (MNRJ 3897), *Helicops carinicaudus* (IVB 834, 926), *Oxyrhopus petolarius digitalis* (IVB 3430), *Oxyrhopus clathratus* (ZUEC 1592), *Philodryas olfersii olfersii* (IVB 1761), *Philodryas patagoniensis* (IVB 3388); *Sibynomorphus neuwiedi* (IVB 591, 1941), *Thamnodynastes* cf. *nattereri* (IVB 383, 952, 2326), *Xenodon neuwiedii* (IVB 2009, 2337); Colubridae: *Chironius bicarinatus* (IVB 965), *Chironius exoletus* (IVB 525) *Chironius laevicollis* (IVB 921, 1560), *Mastigodryas bifossatus* (IVB 573, 722), *Pseustes sulphureus poecilostoma* (IVB 3093), *Spilotes pullatus anomalepis* (IVB 3056), *Oxybelis aeneus* (IVB 3225), *Leptophis ahaetulla ahaetulla* (IVB 2851) Elapidae: *Micrurus corallinus* (IVB 3293); Typhlopidae: *Typhlops brongersmianus* (IVB 1903); Viperidae: *Bothrops jararaca* (IVB 3383), *Bothrops jararacussu* (IVB 3339).
